# Patient involvement to inform the design of a clinical trial in postbariatric hypoglycaemia

**DOI:** 10.1186/s12874-020-01171-z

**Published:** 2020-11-30

**Authors:** Matthias Hepprich, Marc Y. Donath, Lars G. Hemkens

**Affiliations:** 1 Division of Endocrinology, Diabetology and Metabolism, University Hospital Basel, Petersgraben 4, 4053 Basel, Switzerland; 2 Clinic of Endocrinology, Cantonal Hospital Olten, Basler Strasse 150, 4600 Olten, Switzerland; 3 Basel Institute for Clinical Epidemiology and Biostatistics, Department of Clinical Research, University of Basel and University Hospital Basel, Basel, Switzerland; 4 Meta-Research Innovation Center Berlin (METRIC-B), Berlin Institute of Health, Berlin, Germany; 5 Meta-Research Innovation Center at Stanford (METRICS), Stanford University, Stanford, CA USA

**Keywords:** Patient involvement, Postprandial hypoglycaemia, Late-dumping, Bariatric surgery, Outcome finding

## Abstract

**Background:**

Bariatric surgery may lead to symptomatic postprandial hypoglycaemia as a major side effect without established therapy so far. We aimed to develop an evidence-based study design of a clinical trial that tests treatment options and can provide useful patient-relevant evidence.

**Methods:**

We searched systematically for guidance of core outcome sets to determine the most relevant types of outcomes and duration of such a trial. Our search comprised literature databases, a database of core outcome sets and self-help organizations. We then developed a simple online questionnaire based on interviews with German-speaking patients with postprandial hypoglycaemia after bariatric surgery. We recruited participants by reaching out to all German speaking endocrinologists in Switzerland and large Swiss bariatric centres. We asked for preferences regarding outcome types and acceptable duration of being included in a corresponding clinical trial.

**Results:**

The literature search did not identify evidence-based guidance for informing our study design. Experience of clinical and research routine as well as patient interviews helped in identifying potential outcomes and the design of an online questionnaire. Therein, a total of 29 persons started the questionnaire and 22 answered questions related to the primary outcome. Of these, 17 (77.3%) deemed quality of life more relevant as primary outcome than the rate of hypoglycaemic episodes. A trial length of four weeks or longer was regarded as acceptable for 19 of 21 respondents to this question (91.4%) and of six months or longer for 12 respondents (56%).

**Conclusions:**

In situations with no other guidance, a simple questionnaire may help to inform trial design decisions. This study identifies a patient preference for “quality of life” as a primary outcome and supports the evidence-based conception of a patient-centred clinical trial in postbariatric hypoglycaemia.

**Supplementary Information:**

**Supplementary information** accompanies this paper at 10.1186/s12874-020-01171-z.

## Background

Bariatric surgery is one of the most effective interventions to achieve long-term weight loss in patients with severe obesity, but frequently leads to serious side effects [1–3]. One of them is postprandial hypoglycaemia (PBH), previously referred to as late-dumping, affecting in standardized test settings about 50% of patients with a gastric bypass [4–6] although it is frequently undiagnosed [7]. This may lead to a reduced quality of life [8], secondary weight gain [9, 10] and may even be associated with an increased mortality [11–13]. Serious hypoglycaemia requiring assistance of third parties and/or hospitalization for hypoglycaemia affects around 1% [8, 14]. Although some patients are able to effectively control hypoglycaemic episodes with dietary modification, there is no established treatment for severely affected patients with frequent postprandial hypoglycaemia [5].

A recent exploratory trial with 12 patients after gastric bypass indicated that both the SGLT2-inhibitor empagliflozin and the IL-1 receptor antagonist anakinra may reduce postprandial insulin release and prevents hypoglycaemia [15]. These findings call for further evaluation in a larger randomized clinical trial.

We aimed to develop an evidence-based study design for such a trial that tests these treatment options and can provide useful patient-relevant evidence on their merits. However, selecting a primary outcome that appropriately reflects the needs of patients turned out to be a major challenge. Ideally, such a trial would explore patient-relevant outcomes but also improve the understanding of the underlying metabolic effects of the tested treatments that would mediate potential patient-centred benefits (such as glucose levels).

Clear frameworks such as those established for assessment of anti-diabetic treatments that may help to utilize hypoglycaemia as trial outcome in non-diabetic persons are lacking. Extrapolation of such frameworks to nondiabetic patients may be inappropriate and would require strong assumptions as changing glucose levels in diabetic and nondiabetic persons may correspond to different symptoms and health effects.

For postprandial hypoglycaemia, no core outcome set (COS), as “an agreed standard set of outcomes that should be measured and reported, as a minimum, in all clinical trials in specific areas of health or healthcare” [16] has been published. In 2016, the BARIAtric and metabolic surgery Clinical Trials (BARIACT) project defined COS for patients undergoing bariatric surgery based on patient and healthcare provider perspectives. However, postprandial hypoglycaemia was not among the list of candidate outcomes included in the delphi processes [17].

We performed a thorough literature search in September 2019 for clinical trials specifically including patients with symptomatic postprandial hypoglycaemia and found only small trials using biomarkers (e. g. time of glucose levels below a specific threshold) [18, 19] or clinical scoring systems (e. g. Edinburgh Hypoglycaemia Scale) mostly within standardized mixed meal test-settings [20–22] which do not reflect real-life circumstances and provide no patient-relevant information. Only one trial that we identified reported effects on quality of life as a secondary outcome [23].

The measurement of hypoglycaemic episodes by continuous glucose measurement systems appears to accurately depict real-life glucose level variation and may give an objective measurement of the metabolic effects of an intervention aiming to alleviate symptomatic metabolic disorders [24]. However, it is unclear how relevant it is for patients with symptomatic hypoglycaemic episodes to measure merely metabolic changes, since some hypoglycaemic episodes may not be recognized [7, 25, 26] and have varying symptomatic presentation, especially when occurring frequently or during the night [7]. Measuring the frequency of hypoglycaemic episodes alone without consideration of their severity and patient-reported symptoms, seems inadequate to reflect patients’ needs. Furthermore, using the classical Whipple’s triad (i.e. appearance of typical symptoms, low plasma glucose, and relief of symptoms following glucose administration) would be unfeasible in a real-life evaluation. We, therefore, sought to identify the outcome parameter that is most relevant for patients and their subjective wellbeing.

## Methods

The aim of this project was to develop an evidence-based study design of a clinical trial that tests treatment options and can provide useful patient-relevant evidence.

First, we searched published articles that might address outcomes for clinical trials on postbariatric hypoglycaemia to identify outcome domains and to obtain a list of potential core outcome domains [27] with searches on PubMed using terms related to bariatric surgery and hypoglycaemia (we searched first in September 2019 and searched again in July 2020; the detailed search strategy is in the Appendix). as well as COMET initiative database (http://www.comet-initiative.org/studies/), and Google scholar using the terms “patient-relevant outcome” or “patient-centered outcome” combined with “bariatric surgery”, “gastric bypass”, “sleeve gastrectomy”,“late-dumping” or “postprandial hypoglycaemia” excluding articles with the term “diabetes”.

Second, we set out to identify specific self-help organizations for patients with postbariatric hypoglycaemia in Switzerland using a google and facebook search with the German terms for “self-help organization” and “bariatric surgery” or “bypass” and “late-dumping” or “hypoglycaemia”.

Third, we approached the patients in our clinic and the Hypo-BEAR trial participants [15] to collect in unstructured discussions with individual patients any potential patient-relevant outcome measures with various aspects of daily life that would be affected by hypoglycemic episodes (s. supplements, preliminary questionnaire). We then presented to two of the most severely affected patients in our clinic with postprandial hypoglycaemia who agreed to support us for this project with their patient feedback to rank each item from 0 (not important at all) to 10 (very important).

Fourth, we developed an anonymous limesurvey® online questionnaire in German on the basis of the previous findings that quality of life and a reduction of hypoglycaemia would be the most relevant outcome domains (s. supplements, short questionnaire [28]).

With this approach, we aimed to prioritize the outcome candiates, according to patients’ preferences and values, and then determine the study duration and follow-up. The questionnaire contained a brief outline of the nature of the study, that persons with confirmed postprandial hypoglycaemia after bariatric surgery can participate and that it is entirely voluntary. The data items and corresponding questions are listed in Tables 2 and 3. On 25 October 2019, the questionnaire was sent via email to all German speaking endocrinologists by the Swiss Society of Endocrinology and Diabetes as well as directly by the study team to large bariatric centres in the German-speaking part of Switzerland (Clarunis Centre for Gastrointestinal and Liver Diseases, St. Clara Hospital and University Hospital, Basel; Cantonal Hospital Aarau; Cantonal Hospital Solothurn Site Olten and Site Solothurn; Centre of Bariatric Surgery, Bern). The healthcare providers then asked their patients to fill out an online version of the questionnaire. Where this was not possible we provided them with a printed version, that could be filled out by hand and was later digitized by our study team.

### Statistical analysis

All analyses were descriptive. We calculated medians and interquartile ranges unless stated otherwise.

### Ethics and transparency

The study was conducted in compliance with the current version of the Declaration of Helsinki, the ICH-GCP or ISO EN 14155 as well as national legal and regulatory requirements and approved by the local ethics committee (EKNZ Req-2019-00933). All data and material are available online in the Open Science Framework under 10.17605/OSF.IO/49RDE.

## Results

The search in PubMed yielded 521 hits, none of them were deemed relevant after screening titles and abstracts (Table 1). The free-text search in Google Scholar and the search on the COMET database also retrieved no pertinent information. We identified two main organizations dealing with bariatric patients – the Swiss Obesity Foundation [29] and the Swiss Bariatric Surgery Self-help Network [30]. Both online plattforms offered contact details to various self-help organizations within Switzerland. None spefically addressed a subgroup for postbariatric hypoglycaemia. Also the internet forum of the Swiss bariatric surgery self-help network did not yield results for “late-dumping” or “hypoglycaemia” nor was it possible to identify a spokesperson for respective patients.

**Table 1:** Study flow listing applied methods with date, findings and respective results

Method	Date	Findings	Results
**Literature search**			
PubMed	09/2019 06/2020	521 titles/abstracts (last search)	no pertinent articles
Google scholar	09/2019	278 titles/abstracts	
COMET database	09/2019	0 hits	
**Search for patient organizations in CH**	09/2019	Swiss Self-help Organization for Bariatric Surgery (www.shg-bern.ch) Swiss Obesity Foundation with 23 self-help organizations (www.saps.ch)	no specific groups or spokesperson on web-plattform and forum for PBH no specific groups or spokesperson for PBH
**Potential outcome collection by clinical and research experience**	10/2019	13 potential outcome parameters	design of a preliminary questionnaire, s. supplements
**Patient interviews/feedbacks from patients using a preliminary questionnaire**	10/2019	two severely affected patients	inconclusive, many items rated as important
**Final online questionnaire**	10/2019–02/2020		s. Table 2 and Table 3

The two severely affected patients directly providing feedback on the list of outcomes resulting from discussions with other patients valued almost all outcome parameters as important. These data confirmed our expectation that more global outcome domains would be preferable. Similar to trials in oncology [31] and as part of the COS of the BARIACT project [17], we selected “quality of life” as one patient-relevant outcome parameter that would encompass multiple identified items. As our second potential outcome, we chose a reduced rate of hypoglycaemic episodes. Both reliably measurable by validated instruments such as questionnaires [8] or continuous glucose monitoring systems respectively [24].

The online questionnaire was active from 8 October 2019 to 26 February 2020. 29 persons accessed the online questionnaire website, 22 responded to the question related to the primary outcome and 20 provided all requested information.

The majority (17 of 21 respondents, 89%) were female and median age was 40.5 years (IQR 31–51.5). Most responders (*n* = 18) underwent Roux-Y gastric bypass surgery, one participant sleeve gastrectomy and another one both (Table 2). The median postoperative interval was five years (IQR 2.8–7). Ten respondents (50%) reported several hypoglycaemic episodes per week, five (25%) reported daily episodes, and five (25%) stated to have hypoglycaemic episodes at least once a month. Nine (45%) reported to have a drug therapy for their postprandial hypoglycaemia of which six respondents specified their therapy (Table 2). One patient received a triple therapy.

**Table 2:** Baseline characteristics from participants of the online questionnaire

Characteristic	Median (IQR; range)	n (%)
**Total**		29 (100%)
**Age**	40.45 (IQR 31–51.5; 20–57)	
**Sex**		
male		2 (6.9%)
female		17 (58.6%)
no response		10 (34.4%)
**Type of surgery**		
Roux-Y gastric bypass		18 (62.1%)
sleeve gastrectomy		1 (3.4%)
others		1 (3.4%)
no response		9 (31.0%)
**Duration since bariatric surgery (years)**	5 (IQR 2.8–7; range 1–9)	
**Number of hypoglycemic episodes per week**		
daily		5 (17.2%)
not daily, but several days a week		10 (34.5%)
not weekly, but at least once a month		5 (17.2%)
no response		9 (31.0%)
**Patients receiving off-label drug therapy**		
yes		9 (31.0%)
no		11 (37.9%)
no response		9 (31.0%)
**Type of off-label therapy for postbariatric hypoglycemia**		
acarbose		2
liraglutide		2
empagliflozin		2
octreotide		1
onglyza		1

Of all responding participants, 17 (77.3%) deemed quality of life more relevant as primary outcome than the rate of hypoglycaemic episodes (5 participants; 22.7%; Table 3). Of 21 responders, 19 (91.4%) found taking a study medication for a period of four weeks acceptable, 15 (71.3%) would accept three months, while only 12 (52.1%) found six months or longer acceptable. Six patients (28.5%) voted for a trial length of nine months or more (one free text answer opted for 18 months, s. Table 3).

**Table 3:** **Online questionnaire results**

Question	n (%)
**Total**	29 (100%)
**What should a new treatment mainly improve?**	
Quality of life	17 (59.2%)
Less hypoglycemic episodes	5 (18.2%)
no response	7 (22.2%)
**For which trial length could you imagine to take a study medication?**	
two weeks	2 (9.5%)
one month	4 (19.0%)
three months	3 (14.3%)
six months	6 (28. 5%)
nine months	0 (0%)
one year	5 (23.8%)
free text option: one and a half year	1 (4.7%)
no response	6 (28.5%)

## Discussion

We used an evidence-based approach to design a clinical trial to test a novel treatment in patients with postprandial hypoglycaemia after bariatric surgery. This informed our decision to focus on quality of life as primary outcome, measured between four weeks and six months after randomization.

Neither our literature search in several databases, nor search for patient organizations provided us with the required information. Since we did not want to rely only on our perspectives as clinicians and clincial researchers we aimed to inform our decisons by involving patients, fully acknowledging that this simple approach cannot replace a sufficiently large and broad endeavour aiming to create a core outcome set, which would be most desireable. Instead, we used a pragmatic approach where we sought to involve patients as early as possible in the conception of our trial. We collected potential outcome candidates during clinical routine and research visits with affected patients. The discussion with two severely affected patients revealed that most of the items were valued as important hampering our approach to narrowing down potential candidates to a more practical number. We chose quality of life as one potential outcome parameter since this would cover many of the preliminary items and is a commonly used valid outcome parameter in clinical trials as well as part of the BARIACT COS [17].

We developed an online questionnaire that was easy to follow and quick to fill out. From a technical perspective the rate of hypoglycaemic episodes might be a most preferred primary outcome because of the objective and reliable measurement by continuous glucose monitoring, providing a clean measurement of physiological effects. However, this would disregard patient-relevant aspects such as inconveniences to take a drug, side effects and be not really patient important as it would not well reflect how patients “feel and function”. Our study showed a clear patient preference of “quality of life” over a “reduced rate of hypoglycaemic episodes”.

Most of the patients expressed a preference for an active trial length (i.e. intake of study medication) of four weeks or even longer, possibly indicating high feasibility and a high willingness of participants to adhere to a longer study period that might be required to show sustained effects of a treatment. Most of the respondents (75%) are strongly affected with hypoglycaemic episodes occurring either daily or several times per week, indicating an urgent need for treatment options. The number of patients that already receive an off-label drug therapy (45%) and still notice a high frequency of hypoglycaemic episodes indicates not only the need for an effective treatment but also highlights problems related to off-label prescribing, such as reimbursement aspects.

Several limitations need to be considered. First, our sample of patients was small and included only German-speaking participants from Switzerland, potentially limiting the generalizability of results. However, the sociodemographic and medical characteristices of the respondents of the questionnaire are very similar to the expected spectrum of patients with postbariatric hypoglycaemia with a female preponderance, younger age, bariatric surgery, and a typical postoperative interval [5, 6, 32]. Second, our preselection of outcome parameters may have excluded other relevant outcomes which more extensive approaches (such as discrete choice experiments or Delphi rounds [33–35]) may reveal. Third, selection bias may have affected our findings. The exact response rate is unknown since we relied on the cooperation of other healthcare providers and that the questionnaire was fully anonymous and voluntary. However, strongly affected patients may have more likely responded and completed the questionnaire due to a higher motivation for finding an effective treatment. Fourth, our questionaire was online which may have introduced barriers to participate for certain patients, but in every centre patients were also offered paper-based forms to increase the number of responders as much as possible.

## Conclusions

Quality of life seems to be the most important outcome for patients with postprandial hypoglycaemia after bariatric surgery. Using quality of life instead of rate of hypoglycaemia as the primary outcome in randomized clincal trials that test treatments for patients with this condition, may provide more useful and patient-relevant evidence to guide health care decisions.

### Supplementary Information

**Additional file 1: Supplementary file 1**. Preliminary Questionnaire English final.

**Additional file 2: Supplementary file 2**. Short Questionnaire for Patient involvement with PBH V1.0.

## Data Availability

The datasets used and/or analyzed during the current study are available from the corresponding author on request.

## Appendix

### Search strategy for PubMed (date of last search 25 June 2020)

(“Bariatric Surgery”[Mesh:NoExp] OR “Gastric Bypass”[Mesh] OR bariatric surg*[Tiab] OR Metabolic Surg*[tiab] OR “gastric bypass”[Tiab] OR “Roux-en-Y”[tiab] OR “Roux en Y”[tiab] OR “sleeve gastrectomy”[Tiab]) AND (“Postgastrectomy Syndromes”[Mesh] OR “late-dumping” [Tiab] OR “Hypoglycemia”[Mesh:NoExp] OR “hypoglycemia”[Tiab] OR “hypoglycaemia”[tiab] OR “late dumping”[Tiab]) NOT (“animals”[MeSH Terms] NOT “humans”[MeSH Terms])

## Abbreviations

COS: core outcome set
IL-1: interleukin 1
PBH: postbariatric hypoglycaemia
SF-36: survey form-36
SGLT2: sodium-glucose like transporter 2
