# Supplementary material for: Patient involvement to inform the design of a clinical trial in postbariatric hypoglycaemia
Source: BMC Med Res Methodol. 2020 Nov 30;20:290. doi: 10.1186/s12874-020-01171-z (PMC7706264; doi:10.1186/s12874-020-01171-z)
Supplement: Supplementary file 1 — Additional file 1: Supplementary file 1. Preliminary Questionnaire English final. [file 12874_2020_1171_MOESM1_ESM.pdf]

Preliminary Items for Patient Involvement for Research in the Treatment of Postprandial Hypoglycemia

Dear Sir or Madam,

We, a team at the Endocrine Outpatient Clinic at the University Hospital Basel and the Department of Clinical Research at the University of Basel, would like to ask for your support.

Our research interests are episodes of low glucose levels (hypoglycemia) after eating (postprandial) in patients after bariatric surgery, i.e. surgery helping people lose weight, such as gastric bypass surgery, sleeve gastrectomy, biliopancreatic diversion or gastric banding. These hypoglycemic episodes are also known as late-dumping and can present in different ways. Symptoms may range from fatigue, lack of concentration, hunger, cold sweat, nausea or a generally feeling unwell.

So far, there are only dietary measures and no approved medical therapy exists, to treat patients with this condition. We are interested in testing a new therapy. For this, it is necessary to know the needs of patients with this condition. We would like to know what would be the most important measure for a successful treatment of these late-dumping (postprandial hypoglycemia).

Please, do only go through this questionnaire when you had a bariatric surgery (see above) and **you** suffer from late-dumpings (postprandial hypoglycemia). This questionnaire is fully anonymous and voluntary. No conclusions can be drawn about you based on the data collected. You can stop this questionnaire at any time but a complete questionnaire is highly appreciated. Completing the whole questionnaire will take only five minutes. The study was approved by the Ethic Committee Northwest- and Central-Switzerland (EKNZ Req-2019-00933).

1. Please rate each item on a scale from 0 to 10 (0 = least important, 10 = most important).

I value most…

a) … to be cured.

b) … to have a better quality of life.

c) … to be fit for work again/…to be able to work again.

d) … not to have to worry about low blood sugar/hypoglycaemia.

e) … to have fewer hypoglycemic episodes per day/week/month.

f) … having to perform fewer or no blood glucose level measurements.

g) … not to have to worry about getting a good night’s sleep.

h) … being able to exercise without restrictions.

i) … to have no side effects of the medication if possible.

j) … to have low treatment expenses.

k) … to have no additonal doctors apppointments/visits to the clinic.

l) … not having to take medication for all my life.

m) … to be able to become a parent despite/even with medication.

To assess the treatment response sufficiently, long treatment periods may be necessary. This can be a challenge for study participants, but also for the study team.

2. How long would you be willing to participate in a study?

a) 1 month
b) 3 months
c) 6 months
d) 1 year

3. Should there be a compensation for the participation in such a study?
O Yes
O No
O No, but travel cost and other expenses should be covered.

If there is anything else that comes to your mind, please let us know.

Thank you very much for your support.

Sincerely,

Your Study Team

Prof Marc Y. Donath, MD
Head of
Clinic of Endocrinology, Diabetes and Metabolism
University Hospital Basel

Matthias Hepprich, MD
Scientific Staff

Lars Hemkens, MD, MPH
Clinical Epidemiology
Department of Clinical Research
