# Supplementary material for: Patient involvement to inform the design of a clinical trial in postbariatric hypoglycaemia
Source: BMC Med Res Methodol. 2020 Nov 30;20:290. doi: 10.1186/s12874-020-01171-z (PMC7706264; doi:10.1186/s12874-020-01171-z)
Supplement: Supplementary file 2 — Additional file 2: Supplementary file 2. Short Questionnaire for Patient involvement with PBH V1.0. [file 12874_2020_1171_MOESM2_ESM.pdf]

Universitätsspital Basel

Questionnaire for Patient Involvement for Research in the Treatment of Postprandial Hypoglycemia

Dear Sir or Madam,

We, a team at the Endocrine Outpatient Clinic at the University Hospital Basel and the Department of Clinical Research at the University of Basel, would like to ask for your support.

Our research interests are episodes of low glucose levels (hypoglycemia) after eating (postprandial) in patients after bariatric surgery, i.e. surgery helping people lose weight, such as gastric bypass surgery, sleeve gastrectomy, biliopancreatic diversion or gastric banding. These hypoglycemic episodes are also known as late-dumping and can present in different ways. Symptoms may range from fatigue, lack of concentration, hunger, cold sweat, nausea or a generally feeling unwell.

So far, there are only dietary measures and no approved medical therapy exists, to treat patients with this condition. We are interested in testing a new therapy. For this, it is necessary to know the needs of patients with this condition. We would like to know what would be the most important measure for a successful treatment of these late-dumping (postprandial hypoglycemia).

Please, do only go through this questionnaire, when you had a bariatric surgery (see above) and **you** suffer from late-dumpings (postprandial hypoglycemia). This questionnaire is fully anonymous and voluntary. No conclusions can be drawn about you based on the data collected. You can stop this questionnaire at any time but a complete questionnaire is highly appreciated. The whole questionnaire will take only five minutes. The study was approved by the Ethic Committee Northwest- and Central-Switzerland (EKNZ Req-2019-00933).

1. What should a new treatment mainly improve?

- Quality of life
- Less hypoglycemic episodes
- no response

2. For which trial length could you imagine taking a study medication?

- two weeks
- one month
- three months
- six months
- nine months
- one year
- free text option: _____
- no response

Please provide the following general information about yourself.**

- **Age** (years)*______
- **Sex**
  - male
  - female
  - no response

- **What bariatric procedure was performed?***
  - Gastric Sleeve
  - Gastric Bypass
  - other: ______________ (please specify)
- **How many years have passed since your bariatric surgery?** (years)* _______
- **How often do you experience postprandial hypoglycemic episodes?**
  - Every day
  - Not every day, but several times a week
  - Not every week, but several times a months
  - No response
- **Do you receive a medication against postprandial hypoglycemia?**
  - No
  - If yes, please specify: ______________

* mandatory

If you can think of anything else, please let us know?

Many thanks for your support. Sincerely,

Your Study Team

Prof Dr Marc Y. Donath, MD
Head of
Clinic of Endocrinology, Diabetes and Metabolism
University Hospital Basel

Dr Matthias Hepprich, MD
Scientific Staff

PD Dr Lars Hemkens, MD, MPH
Clinical Epidemiology
Department of Clinical Research
University of Basel
